# Evaluating a communication aid for return of genetic results in families with hypertrophic cardiomyopathy: A randomized controlled trial

**DOI:** 10.1002/jgc4.1651

**Published:** 2022-11-17

**Authors:** Charlotte Burns, Laura Yeates, Joanna Sweeting, Christopher Semsarian, Jodie Ingles

**Affiliations:** ^1^ Agnes Ginges Centre for Molecular Cardiology at Centenary Institute The University of Sydney Sydney Australia; ^2^ Faculty of Medicine and Health The University of Sydney Sydney Australia; ^3^ Department of Cardiology Royal Prince Alfred Hospital Sydney Australia; ^4^ Cardio Genomics Program at Centenary Institute The University of Sydney Sydney Australia

**Keywords:** communication, genetic counseling, genetic testing, inherited heart disease

## Abstract

Genetic testing for hypertrophic cardiomyopathy (HCM) is considered a key aspect of management. Communication of genetic test results to the proband and their family members, can be a barrier to effective uptake. We hypothesized that a communication aid would facilitate effective communication, and sought to evaluate knowledge and communication of HCM risk to at‐risk relatives. This was a prospective randomized controlled trial. Consecutive HCM patients attending a specialized clinic, who agreed to participate, were randomized to the intervention or current clinical practice. The intervention consisted of a genetic counselor‐led appointment, separate to their clinical cardiology review, and guided by a communication booklet which could be written in and taken home. Current clinical practice was defined as the return of the genetic result by a genetic counselor and cardiologist, often as part of a clinical cardiology review. The primary outcome was the ability and confidence of the individual to communicate genetic results to at‐risk relatives. The a priori outcome of improved communication among HCM families did not show statistically significant differences between the control and intervention group, though the majority of probands in the intervention group achieved fair communication (*n* = 13/22) and had higher genetic knowledge scores than those in the control group (7 ± 3 versus 6 ± 3). A total of 29% of at‐risk relatives were not informed of a genetic result in their family. Communication among HCM families remains challenging, with nearly a third of at‐risk relatives not informed of a genetic result. We show a significant gap in the current approach to supporting family communication about genetics. Australian New Zealand Clinical Trials Registry: ACTRN12617000706370.

**What is known about this topic**

Communication between family members regarding genetic testing can be a barrier to effective uptake.

**What this paper adds to the topic**

Communication is a challenge in HCM families, with nearly a third of at‐risk relatives not informed of a genetic result. There is a significant gap in the current approaches to supporting family communication about genetics.

## 1 INTRODUCTION

Hypertrophic cardiomyopathy (HCM) is a clinically heterogeneous disease characterized by unexplained left ventricular hypertrophy in the absence of a loading condition such as hypertension (Maron et al., 2012). HCM affects at least 1 in 500 with some being relatively asymptomatic, while others experience more severe symptoms such as heart failure requiring transport, or sudden cardiac arrest/death (Semsarian et al., 2015). HCM genetic testing can allow identification of a causative genetic variant, which can be used for cascade genetic testing and clarifying risk status of family members (Ingles et al., 2019). Effective pre‐test and post‐test genetic counseling is critical and recognized in disease guidelines, and focuses on clear communication of the likely outcomes of HCM genetic testing (Burns et al., 2018; Ommen et al., 2020).

Genetic counseling is important for families with HCM, not just in supporting genetic testing including explaining uncertain and complex results, but also for understanding inheritance risks, characterization of the family history, information and psychosocial support (Ingles et al., 2011). In the clinic setting, pre and post‐test genetic counseling include discussion of inheritance risks to family members and clinical screening recommendations, allowing asymptomatic at‐risk relatives to make pre‐emptive, educated decisions concerning their own likelihood of developing disease and how that may impact future family planning. Understanding how a patient interprets and conveys this genetic information to their at‐risk relatives is critical to ensuring patients' get the most value out of genetic testing. Broader family communication relies on the proband and several studies highlight the many ways this can be problematic, including lack of retention or understanding of the information presented (Burns et al., 2016; Burns, Yeates, et al., 2017;Patenaude et al., 2013; Young et al., 2017).

It is estimated 20%–40% of at‐risk relatives are unaware of pertinent genetic information and even when informed of their own risk, do not act on this knowledge. (Burns et al., 2016; Christiaans et al., 2008; Gaff et al., 2007). Many patients report that uncertain genetic results are conveyed even less frequently among families (Burns, Yeates, et al., 2017). Evidence from cancer genetics research shows risk awareness and understanding of results, though wide‐ranging, can be poor, inaccurate and incomplete (Patenaude et al., 2013; Young et al., 2017).

Decision or communication aids are tools specifically designed to support decision‐making and unmet information needs and are effective in improving knowledge and accuracy of risk perceptions (Christian et al., 2021; Stacey et al., 2014; Wakefield et al., 2007). A growing number of studies are beginning to address interventions to facilitate effective communication with relatives at risk of inherited cardiac diseases (Christian et al., 2021; Harris et al., 2019; van den Heuvel et al., 2019). We hypothesize that improving knowledge of an HCM genetic result would have a positive impact on communication to at‐risk relatives. We aim to evaluate knowledge and communication of HCM risk to relatives following use of a custom designed HCM decision aid. The decision aid has been previously developed and trialed to demonstrate feasibility and acceptability (Smagarinsky et al., 2017) and the protocol has been published (Burns et al., 2019).

## 2 METHODS

### 2.1 Study design

**FIGURE 1:**
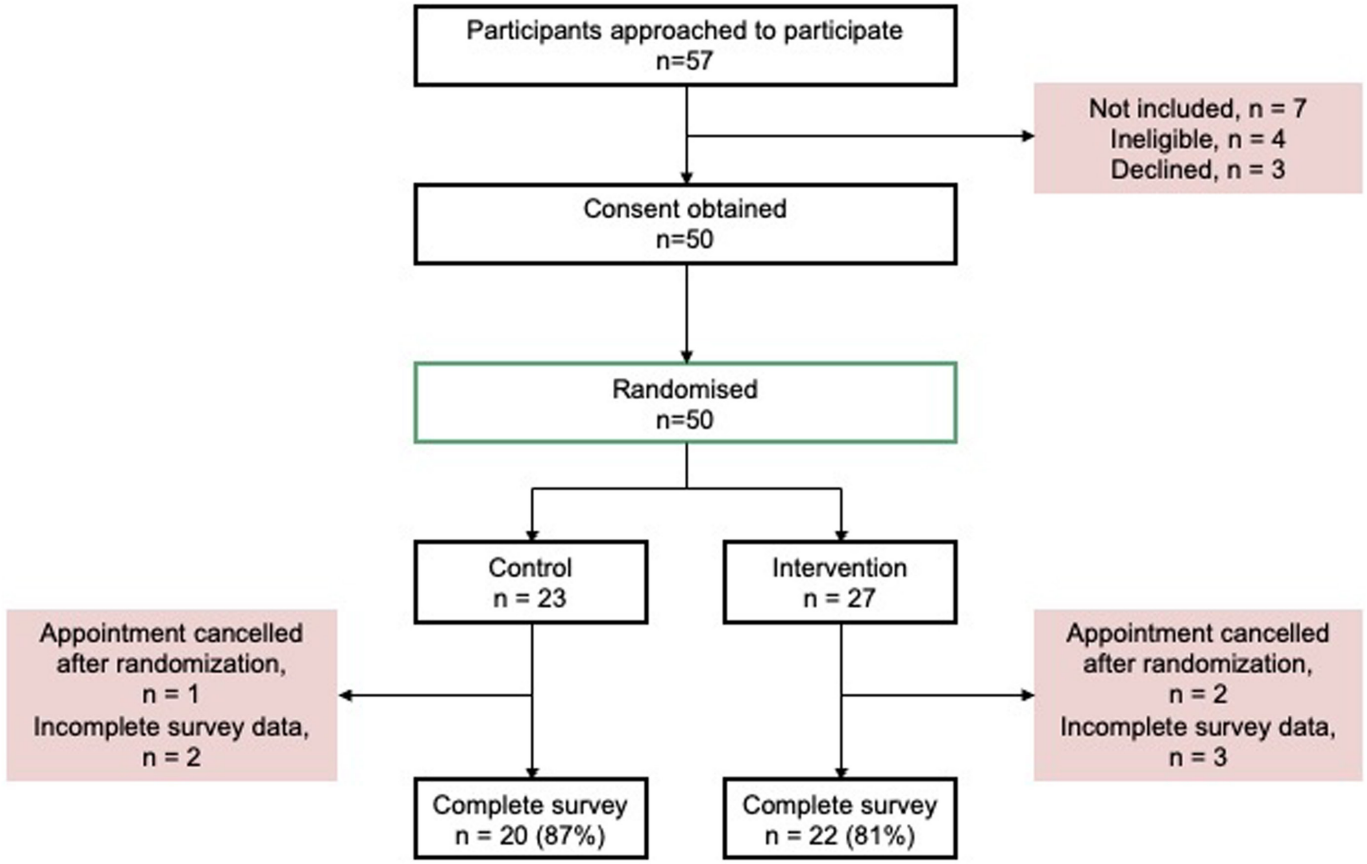
Flowchart of randomized controlled trial study probands

This was a prospective randomized controlled trial (Burns et al., 2019). The trial was registered with the Australian New Zealand Clinical Trials Registry: ACTRN12617000706370. Consecutive HCM patients were invited to participate during their genetic counseling intake call to notify them that their genetic result was ready to be returned. After verbal consent was attained, participants were randomized to either the intervention or control arm of the study to receive their genetic result (Figure 1). The local Human Research Ethics Committee approved the study (Sydney Local Health District Ethics Review Committee; X16‐0030).

### 2.2 Participants

This trial was conducted in a specialized multidisciplinary HCM clinic at the Royal Prince Alfred Hospital, Sydney, Australia. Eligible participants included HCM probands with a genetic result ready for return, those aged 18 years or older and with sufficient written English skills. Genetic testing was performed either via a research study or by a clinically accredited laboratory as previously described (Burns, Bagnall, et al., 2017). Variants were classified using the *MYH7*‐modified ACMG/AMP criteria as per our usual clinical practice (Kelly et al., 2017). Recruitment commenced in November 2017 and was completed in November 2018. Participants were invited during the genetic counseling pre‐clinic intake phone call conducted as per our regular clinical process, with participant verbally indicating their interest. Written informed consent was obtained. All participants received pre‐test genetic counseling by experienced genetic counselors as part of their routine clinical care.

### 2.3 Randomization

The Excel (Microsoft Office) “Random” function was used to generate a randomized list with study participants assigned the next number on the random list. This number was linked to either control or intervention. The randomization was performed by a researcher not connected to the study to ensure allocation concealment.

### 2.4 Sample size and power calculations

A priori sample size calculations were performed based on results from our published feasibility study (Smagarinsky et al., 2017). The primary outcome of this trial was the ability and confidence of the proband to communicate genetic results to at‐risk relatives. Data from the feasibility study indicated 75% of participants communicated genetic results to at‐risk relatives. We conservatively assumed the control group would communicate in 50% of cases, at a significance level of 5% and 80% statistical power, giving a sample size of *n* = 21 required per group. We planned to recruit additional patients with the aim of reducing the impact of drop‐out and incomplete survey data on endpoints.

### 2.5 Communication aid development

The communication aid was developed to support the delivery of genetic results to the proband and to aid communication with the family. The communication aid published and reported in the pilot study by Smagarinsky et al was used in this current randomized controlled trial (Smagarinsky et al., 2017). The pilot data demonstrated feasibility and acceptability of the communication aid.

### 2.6 Control group

Those in the control group received their result as per current clinical practice. In our practice, this typically involved return of a genetic result by the cardiologist and genetic counselor (CB, LY, JI, CS), usually following clinical review which was often the primary purpose of the appointment. In the majority of cases a genetic counselor was present.

### 2.7 Intervention group

Those randomized to the intervention were allocated an additional appointment time following the clinical review consultation with their cardiologist. In this appointment, they saw the cardiac genetic counselor (CB and JI) who returned their genetic result using the communication aid.

The communication aid is a booklet outlining the process of genetic testing and consequences of a genetic result for at‐risk relatives (Burns et al., 2019). Each category of genetic result is discussed, including indeterminate (no variant identified), a variant of uncertain significance and a likely pathogenic or pathogenic, actionable result. During discussion with the genetic counselor, the relevant category of result and associated recommendations for the family were highlighted.

### 2.8 Data collection and outcomes

Primary and secondary outcomes were measured using a survey at a single time point (two‐week post intervention). The survey contained previously published and validated scales along with demographic questions. The survey was administered online via Qualtrics (https://www.qualtrics.com/) with participants receiving a direct link. Hard copies with a return‐paid envelope were provided where requested. Evidence is sparse regarding the most appropriate time lapse between genetic result disclosure and family communication. Two weeks post result disclosure was deemed appropriate by the study team due to the risk of arrhythmia and potential for sudden death in the context of inherited heart disease. Return of the survey was followed up on a fortnightly basis, with a maximum of 2 reminders.

### 2.9 Primary outcome

The primary outcome was the ability and confidence of the proband to communicate genetic results to their at‐risk relatives. This was measured at a single time point, and collected two weeks after return of genetic results. Two measures were used to assess ability and confidence, and then combined to provide a binary outcome. The certainty sub‐scale of the Psychological Adaptation to Genetic Information (PAGIS) scale was used to measure confidence with genetic knowledge (Read et al., 2005). This sub‐scale assesses the patients' awareness and confidence in their genetic knowledge. Consequent ability to convey this information was assessed by the percentage of at‐risk relatives informed of genetic results by the participant. The percentage was calculated by counting the living first‐degree relatives informed of their risk, and dividing by the total number of living first‐degree relatives. Scores from both measures were averaged to determine a final score. The final score was translated to a binary outcome of “fair” versus “poor” ability and confidence to communicate genetic results to at‐risk relatives.

Fair communication was considered an average score of 75% and over, and poor communication <75%. We came to this cut‐off after review of the literature and determined that communication rates fall between 60–80% but more often below 75% (Batte et al., 2015; Christiaans et al., 2008; Ison et al., 2019). In addition, we reviewed data from our previous studies in the field that showed similar rates of non‐communication (Burns et al., 2016).

Factors impacting communication of genetic results to at‐risk relatives are multidimensional. Therefore, we chose a combination approach to more broadly reflect the communication process. Previous studies have depended on single and linear measures of communication such as contact by family members with the genetics department or self‐reported communication with at‐risk relatives only. To overcome this limitation, the study was designed to combine the proband's confidence regarding knowledge of genetics with the action linked to this knowledge, i.e., consistency between the probands confidence with their genetic information in combination with their self‐reported percentage of immediate family members informed.

The certainty sub‐scale of the PAGIS was used. This measures confidence with genetic knowledge (Read et al., 2005). The PAGIS incorporates the multidimensional adaptation to genetic information and comprises five domains which include; (a) non‐intrusiveness, (b) support, (c) self‐worth, (d) certainty and (e) self‐efficacy. Evidence for the utility of this scale has been published and illustrates its potential use for assessing genetic counseling interventions (Read et al., 2005).

### 2.10 Secondary outcomes

Secondary outcomes were assessed via three additional scales, a series of questions regarding communication with relatives, in addition to a number of demographic questions.

An amended version of the Breast Cancer Genetic Counseling Knowledge Questionnaire (BGKQ) was used to assess genetic knowledge (Erblich et al., 2005). It measures understanding of information related to genetic counseling for breast cancer. The original scale included 27 items, including statements related to genetics such as ‘*50% (half) of your genetic information was passed down from your mother’* with respondents asked if the statement was true or false. The original scale was empirically derived from detailed content analysis of breast cancer genetic counseling sessions. We used 10 items from the original scale, edited to reflect the HCM context.

The Satisfaction with Genetic Counseling Scale (SGCS) was used to assess satisfaction with services received (Shiloh et al., 1990). The original survey measured three areas of patient satisfaction: instrumental, affective and procedural. The procedural dimensions (three questions) were removed.

The genetic counseling outcome scale (GCOS‐24) was used to measure patient reported outcomes of genetic counseling (McAllister et al., 2011). The survey was designed to be used pre‐ and post‐genetic counseling, though we have used it in the post‐counseling setting to compare the control and intervention groups. The scale measures the construct of empowerment to summarize the patient derived benefits from genetic counseling, with a high score is indicative of patients feeling empowered with the information received in a genetic counseling session.

### 2.11 Data analysis

Data were analyzed using Prism (version 7.0) and SPSS (Version 23.0). The primary outcome as a binary measure was compared between the intervention and control group. We used chi‐square analyses using *p* < 0.05 for statistical significance. Published scoring protocols for the validated scales for genetics knowledge, satisfaction with services and genetic counseling outcomes were used in the assessment of secondary outcomes. Mean scores for each scale were compared between the intervention and control group, and comparisons between the control and intervention group were analyzed using unpaired t‐tests for continuous data and chi‐square analysis for categorical data. Sub‐group analyses were performed; specifically, we compared outcomes in the study groups stratified by the gene result [informative (pathogenic/likely pathogenic)] and [uninformative (uncertain or indeterminate)]. We also stratified probands by the presence or absence of a family history of disease.

## 3 RESULTS

### 3.1 Cohort characteristics

We approached 57 eligible HCM probands with a genetic result ready to be returned. This included informative results (pathogenic/likely pathogenic) and uninformative results (variant of uncertain significance and indeterminate). Of those 57 probands, four were deemed ineligible due to (self‐reported) insufficient English language skills and three declined. Fifty probands provided verbal consent to be randomized to the study. After randomization, three probands canceled their clinic appointment. An additional five probands had insufficient data in their surveys to be included in analysis. This included three probands from the intervention arm and two probands from the control arm of the study. In total, there were 20 probands in the control arm and 22 in the intervention arm (Figure 1).

Clinical and demographic characteristics of the probands included in the study are documented in Table 1. There were no statistically significant differences between probands in the control versus the intervention arms. Fifty percent (50%) of probands in the intervention arm and 50% of probands in the control arm had pathogenic/likely pathogenic variants identified. These figures accurately reflect the expected clinical yield of HCM genetic testing. The percent of at‐risk first‐degree relatives informed of a genetic result in the family was 71% (range: 0–100). This indicates 29% of at‐risk relatives were not informed of a genetic result. Communication rates did not differ greatly between control and intervention participants (70% versus 72%). The mean percentage of total at‐risk first‐degree relatives informed of their family members' diagnosis of HCM was 83% (range: 0–100), indicating 17% were not informed.

**TABLE 1:** Participant characteristics

	Intervention	Control	*p*‐value
*N*	22	20	‐
Male gender (%)	16 (73)	18 (90)	0.24
Current age (years)	52 ± 16	50 ± 15	0.61
European ethnicity (%)	19 (86)	14 (70)	0.27
Comorbidities present (%)	4 (18)	6 (30)	0.48
Pathogenic/likely pathogenic variant (%)	11(50)	10 (50)	1
ICD in situ (%)	6 (23)	9 (45)	0.23
SCD event (%)	2 (9)	1 (5)	1
Family history of clinical disease	10 (45)	6 (30)	0.30
Family history of SCD	3 (14)	3 (15)	1
Number living first‐degree relatives (> 18 yrs)	4.5 ± 2	3.5 ± 2	0.10

Abbreviations: ICD, implantable cardioverter defibrillator; SCD, sudden cardiac death.

### 3.2 Primary outcome

**FIGURE 2:**
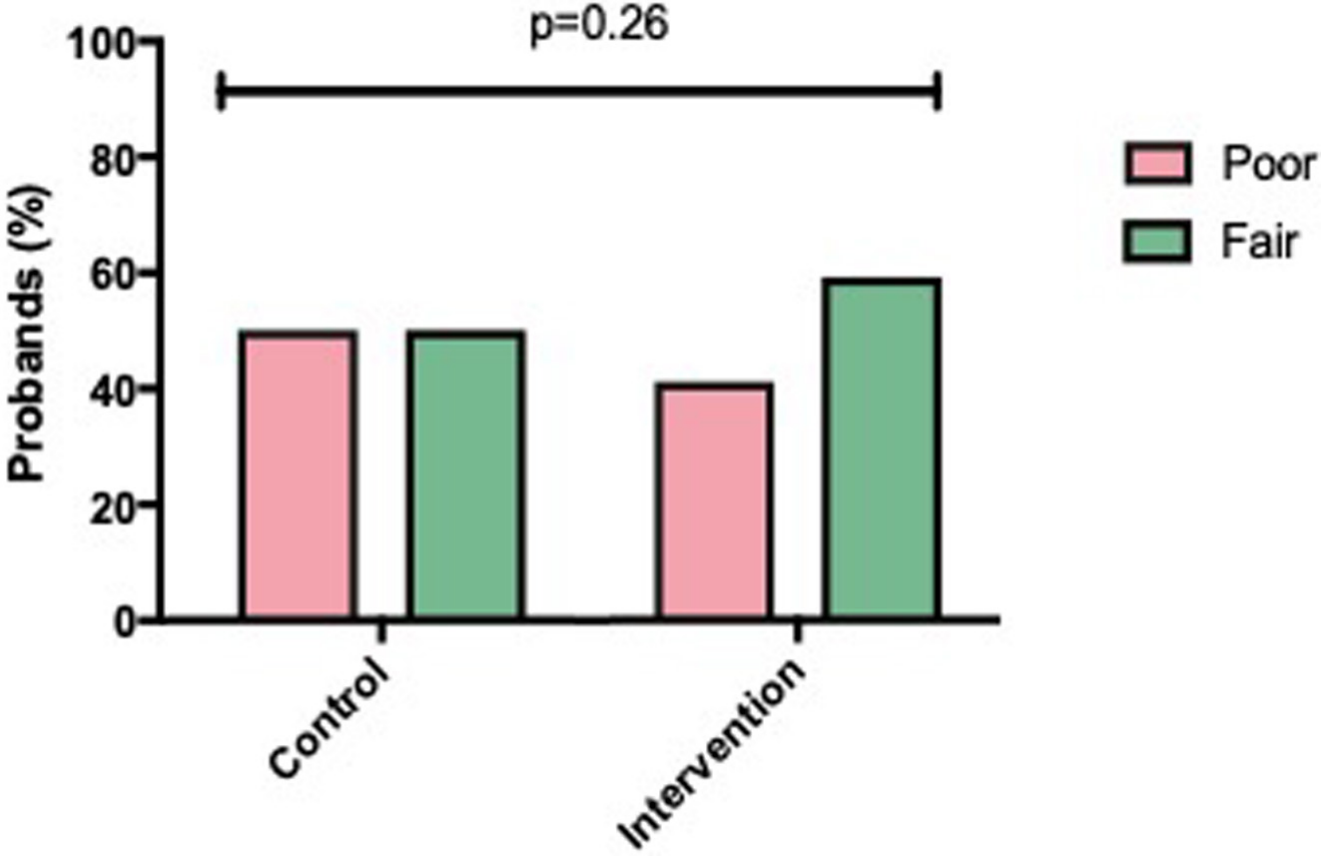
A priori primary outcome. Fair versus poor communication in control and intervention groups. The percentage of probands with fair versus poor communication in the control and intervention groups

The a priori primary outcome measure was an average score which incorporated the certainty sub‐scale from PAGIS and the number of first‐degree relatives informed of their genetic test result. This was a binary score. Though more than half of participants in the intervention group demonstrated “*fair*” communication (≥75%) there was no statistically significant difference between the two groups (intervention: 13/22 [59%] versus control: 10/20 [50%], *p* = 0.26) (Figure 2). In addition, we compared the mean primary outcome score as a continuous variable and found no significant differences between the control and intervention groups (72% ± 4 versus 73% ± 4, *p* = 0.88).

### 3.3 Secondary outcomes

#### 3.3.1 Genetic knowledge

**FIGURE 3:**
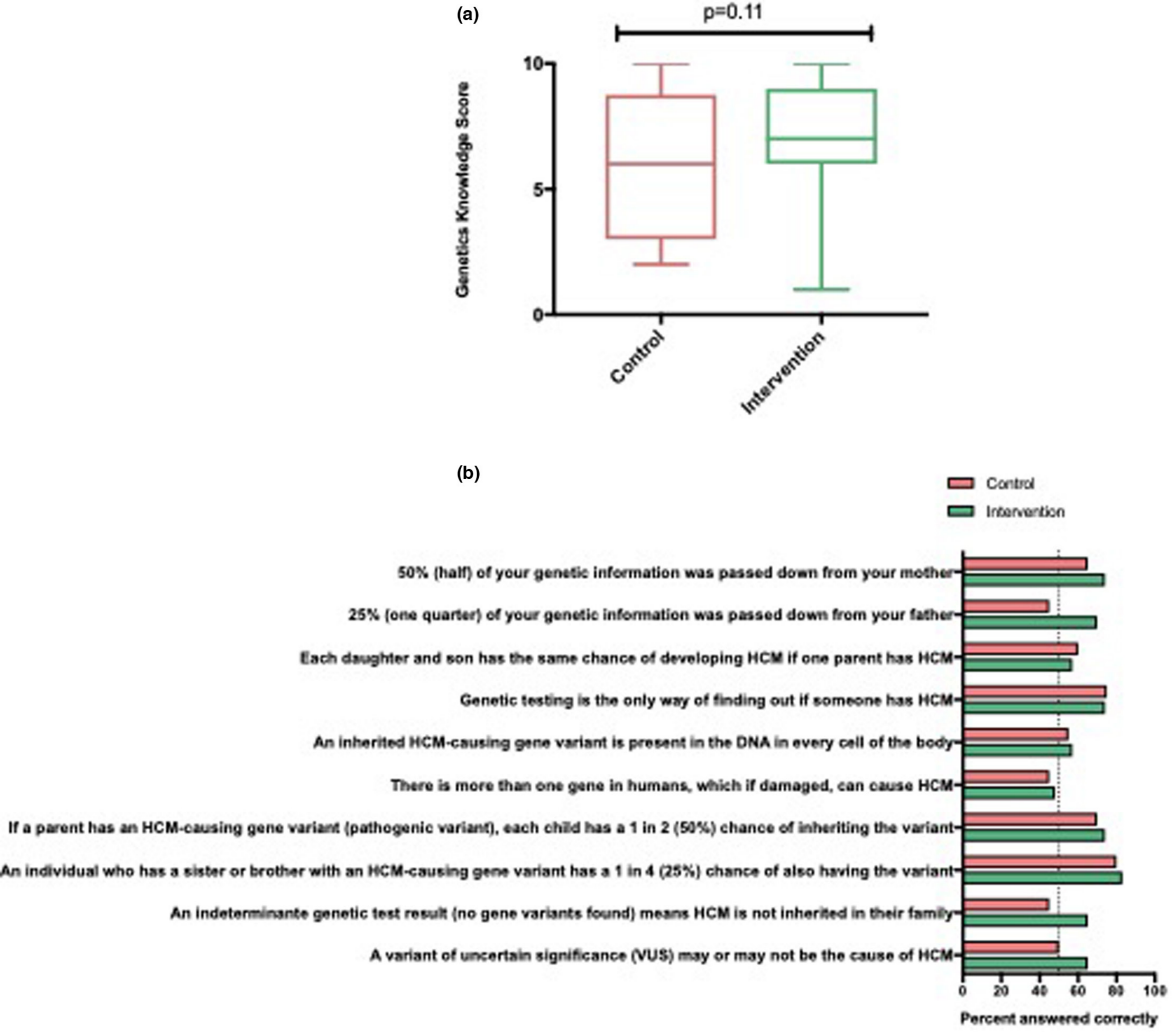
(a) *t*‐test of genetic knowledge scores (maximum score of 10) between probands in the control and intervention groups. (b) Percentage of probands who gave correct answers for genetic knowledge items. Items from the genetic knowledge score answered correctly by control and intervention groups. Abbreviations: DNA, deoxyribonucleic acid; HCM, hypertrophic cardiomyopathy

The mean score for genetic knowledge among the total group was 6/10 (60%). Across the survey items, there were 107/420 ‘do not know’ responses which were scored as incorrect (Figures 3a,b). There was no difference between probands in the intervention and control groups (Table 2). When considering the genetic knowledge score as a pass or fail, i.e., a score of less than 50% was considered a “fail” and >50% a “pass”, more of the intervention group received a pass for genetics knowledge though this did not reach statistical significance.

**TABLE 2:** Secondary outcomes

Measure	Intervention	Control	*p* value
Genetic knowledge (raw score)	7 ± 3	6 ± 3	0.11
Number with ‘Pass’ score (>50%)	19/22 (86%)	12/20 (60%)	0.05
PAGIS	115 ± 16	112 ± 15	0.48
Satisfaction with services	33 ± 6	33 ± 4	0.91
Instrumental	11 ± 2	11 ± 1	0.90
Affective	11 ± 2	11 ± 1	0.76
GCOS‐24	120 ± 15	119 ± 16	0.72
Understanding of need for referral	6.7 ± 0.5	5.6 ± 1.0	0.01

Abbreviations: GCOS‐24, Genetic Counseling Outcomes Scale; PAGIS, Psychological Adaptation to Genetic Information.

#### 3.3.2 Psychological adaptation to genetic information

Overall, the mean total PAGIS score was 114 ± 16 (maximum score of 156) with higher total scores indicating more positive psychological adaptation among the group. When comparing the mean total score between the intervention and control groups, there were no statistically significant differences (Table 2).

The mean total scores for the sub‐scales included: non‐intrusiveness 4.1 ± 0.8 (maximum weighted score of 6), support 4.5 ± 0.8 (maximum weighted score of 6), self‐worth 4.4 ± 1.3 (maximum weighted score of 6) and self‐efficacy 4.2 ± 1 (maximum weighted score of 6). The mean score for certainty was 4.4 ± 0.5 (maximum weighted score of 6), and was incorporated into the primary outcome calculation as described above. We compared the sub‐scale scores between the intervention and control groups and there were no statistically significant differences.

#### 3.3.3 Satisfaction with services

Overall, all probands reported high levels of satisfaction with the process to return their genetic result. This was indicated by a mean total satisfaction score of 33 ± 5 (maximum score of 36). Higher scores indicate higher levels of satisfaction. For the instrumental and affective components of this scale, mean scores were 11 ± 2 (maximum score of 12) and 11 ± 2 (maximum score of 12), respectively. Single item scores (maximum score of 4) relating to expectations fulfilled, satisfaction with information and overall satisfaction all reflected high levels of satisfaction. When comparing the mean total score between the intervention and control groups there were no statistically significant differences (Table 2). We compared the instrumental and affective components between the intervention and control groups and there were no statistically significant differences (Table 2).

#### 3.3.4 Patient reported outcomes of genetic counseling

The mean GCOS‐24 score was 119 ± 15 (scores range from 24 to 168) which indicates good patient empowerment with higher scores indicating higher levels of empowerment. When comparing the mean score between the intervention and control groups, there were no statistically significant differences (Table 2). Probands in the intervention group were more likely to understand the reasons their doctor referred them to the cardiac genetic service (6.7 ± 0.5 versus 5.6 ± 1.0, *p* = 0.01) (Table 2).

### 3.4 Sub‐group analyses

Sub‐group analyses were conducted by grouping probands into those who received informative (pathogenic/likely pathogenic) and uninformative (VUS/indeterminate) genetic test results. In addition, probands were grouped into those with and without a family history of HCM. There were no differences between the intervention and control groups in the sub‐group analyses.

## 4 DISCUSSION

We describe a randomized controlled trial aimed at investigating the impact of a genetic counselor‐led intervention to return HCM genetic results using a custom designed communication aid. The a priori primary outcome measure for this study was to assess the ability and confidence of the proband to communicate genetic results to at‐risk relatives. Though this did not show statistical significance when compared between the intervention and control group, we highlight some important findings. First, the majority of participants in the intervention group did demonstrate *“fair”* communication as measured by the primary outcome and genetic knowledge scores were consistently higher among the intervention group. In addition, and of great clinical importance, we highlight that up to 29% of at‐risk relatives remained uninformed about a genetic result in their family. Further, up to 17% of at‐risk relatives remain uninformed of the HCM diagnosis itself. This is in spite of the return of results in a specialized multidisciplinary clinic with expertise including experienced cardiac genetic counselors and cardiologists. Uninformed relatives are unable to make proactive decisions regarding their own risk management. Factors influencing communication are multifaceted and likely require more than a single intervention to affect any meaningful improvement.

When asked about family communication, most patients report that families should communicate risk among themselves with varying levels of support from their healthcare providers (Healey et al., 2017; Young et al., 2017). In addition, there is evidence for the effectiveness of genetic counseling to assist with this process (Fiallos et al., 2017; Forrest et al., 2008). In spite of this, the literature consistently demonstrates family communication about genetics is between 60%–80% with a significant number of at‐risk relatives remaining uninformed about their genetic risk (Gaff et al., 2007; Healey et al., 2017). Indeed, a recent randomized controlled trial evaluated a tailored approach to informing at‐risk relatives of their risk of an inherited heart disease, which included the option for a genetic counselor to inform the relatives directly, did not show any significant difference in uptake of genetic counseling at 1 year follow‐up (van den Heuvel et al., 2022). Another study used an online video designed to facilitate family communication about risk of hypertrophic cardiomyopathy, with only modest effectiveness (Harris et al., 2019). Taken together, interventions to improve family communication likely need to be multifaceted to cater to wide ranging family dynamics, health literacy and support needs.

Many factors have been identified which influence family communication about genetic risk. These include complicated family dynamics, guilt, anxiety and gender (Barsevick et al., 2008; Burns et al., 2016; Claes et al., 2003). In addition, the literature and clinical experience highlights loss of contact with relatives and geographically distant relatives is a commonly cited and significant issue (Healey et al., 2017; Young et al., 2017). Importantly, much of this literature comes from the inherited cancer context. Inherited heart diseases have the unique risk of sudden cardiac death, which should be considered when discussing communication of inheritance risk. Initial discussions surrounding a diagnosis of an inherited heart disease are often focused on clinical management of the proband themselves but should highlight the importance of family screening recommendations.

Studies focused on the inherited heart disease patient population aimed at addressing family communication show varying results. One recent study among HCM probands found 80% of first‐degree relatives were informed of their genetic risk with probands acknowledging the unique process of communication for each family but perceiving disclosure of risk information as *‘imperative’* (Hudson et al., 2019). In addition, we have conducted a study in long QT syndrome (LQTS) patients which demonstrated 10% of probands had not disclosed relevant risk information to at least one first‐degree relative (Burns et al., 2016).

Evaluating genetic counseling interventions, particularly related to family communication is difficult. This may account for some of the ambiguity around the best practice approach. There is little agreement about suitable outcome measures to assess the effectiveness of a particular genetic counseling intervention (McAllister et al., 2011; Payne et al., 2008). Here we aimed to address the issue commonly referred to as passive non‐disclosure, whereby relatives intend to disclose and communicate relevant information and do not actively choose non‐disclosure. In spite of this however, communication still does not occur (Gaff et al., 2005). One contributing factor may be the information provided to probands and their knowledge of the appropriate information. Therefore, we aimed for our communication aid to improve knowledge and the information provided regarding family screening. In spite of the time spent with probands during the study, positive satisfaction and outcome scores, good confidence with genetic knowledge alongside a reasonable mean genetic knowledge score (60%), up to 17%–29% of first‐degree relatives among this cohort remain uninformed of their risk.

Essentially all outcomes in the study were non‐significant. We had only short follow‐up time points and design of our intervention may have addressed the wrong aspect of family communication about genetics. As discussed, family communication about genetics is complex and multifaceted, and in choosing to target ability and confidence as important contributors to family communication we may have missed a more appropriate outcome. Interpretation of these results and the literature, highlights the complexity, intricacies and personal and family dynamics that may play a significant role beyond knowledge in the process of family communication. A more tailored approach, addressing individual family needs and drawing upon more tools for supporting communication among families is likely needed.

Nonetheless, overall satisfaction and outcome scores were good. In addition, the cardiac genetic counselors using the communication aid found the aid to be clinically useful and commented that it facilitated the communication of genetic results to probands. In fact, it was identified that returning genetic results without use of the communication aid felt it was lacking after commencement of this study. Though data were not collected systematically, questions raised by the patients during the genetic counseling sessions with the communication aid reflected both a positive experience and firm grasp of the information provided for probands themselves.

## 5 CONCLUSION

We highlight that in spite of satisfaction with services and the information provided, family communication was not improved by the genetic counselor‐led intervention. Complex family dynamics, interpersonal family relationships and the proband's own beliefs about whom they should communicate with all contribute to family communication about genetics. Interventions to support family communication and ensure all at‐risk relatives are appropriately informed will require multifaceted approaches, allowing a tailored offering of support and tools to families.

## AUTHOR CONTRIBUTIONS

**Charlotte Burns:** Conceptualization; data curation; formal analysis; methodology; writing – original draft; writing – review and editing. **Laura Yeates:** Investigation; methodology; writing – review and editing. **Joanna Sweeting:** Formal analysis; writing – review and editing. **Christopher Semsarian:** Investigation; methodology; writing – review and editing. **Jodie Ingles:** Conceptualization; formal analysis; funding acquisition; methodology; project administration; supervision; writing – original draft; writing – review and editing. Authors Charlotte Burns and Jodie Ingles confirm that they had full access to all the data in the study and take responsibility for the integrity of the data and the accuracy of the data analysis. All of the authors gave final approval of this version to be published and agree to be accountable for all aspects of the work in ensuring that questions related to the accuracy or integrity of any part of the work are appropriately investigated and resolved.

## FUNDING INFORMATION

LY is a recipient of a co‐funded National Heart Foundation of Australia / National Health and Medical Research Council (NHMRC) PhD scholarship (#102568/#191351). CS is the recipient of a National Health and Medical Research Council (NHMRC) Practitioner Fellowship (#1154992). JI is the recipient of an NHMRC Career Development Fellowship (#1162929).

## CONFLICT OF INTEREST

JI receives research grant support from Bristol Myers Squibb. All other authors report no competing interests.

## HUMAN STUDIES AND INFORMED CONSENT

Informed consent was provided by the participants of this study. This study was approved by the Ethics review Committee (RPAH Zone) of the Sydney Local Health District (protocol number X16‐0030) and all procedures were followed in accordance with the Helsinki Declaration of 1975, as revised in 2000.

## Data Availability

Additional information and data from this manuscript is available on reasonable request.
